# Supplementary material for: Association between different atherogenic and insulin resistance indices and infertility in polycystic ovary syndrome patients
Source: PLoS One. 2026 May 22;21(5):e0343783. doi: 10.1371/journal.pone.0343783 (PMC13196974; doi:10.1371/journal.pone.0343783)
Supplement: Attachment — Submitted filename: response 2026-01-07.pdf [file pone.0343783.s002.pdf]

Dear Dr. Ricardo Ney Oliveira Cobucci,

We sincerely appreciate the time and effort you and the reviewer have dedicated to evaluating our manuscript, "Association between different atherogenic and insulin resistance indices and infertility in polycystic ovary syndrome patients" (Manuscript ID: PONE-D-25-55841). We are grateful for the constructive feedback, which has helped us improve the quality of our work, and accept our sincerest apologies for the late response, since, because of the total internet shutdown in Iran, we were not able to communicate.

We have carefully addressed all the comments and suggestions provided by the reviewer. Below, we provide a point-by-point response to each remark, detailing the revisions made in the revised manuscript. All changes have been highlighted in the revised version for easy reference in yellow.

Reviewer #1

We are truly grateful for your kind and encouraging words. Your positive feedback means a great deal to our team and reinforces our commitment to further research in this area. Thank you for taking the time to review our work so thoughtfully.

Comment 1: *"TyG-BMI formula in the Methods --> it should be TyGxBMI not TgxBMI."*

Response 1: We corrected this typo.

Comment 2: *"Specify how "unsuccessful conception" was documented and whether other causes of infertility were considered."*

Response 2: Infertility status was determined based on documentation in medical records and defined as failure to achieve pregnancy after at least 12 months of regular unprotected intercourse, as recorded during routine clinical assessments by treating gynecologists. Classification was therefore not based solely on self-report.
Male factor infertility was explicitly excluded. Other female causes of infertility unrelated to PCOS were considered when information was available in medical records; however, infertility was classified as all-cause infertility within a PCOS population. We have clarified this point in the Measurements and Tools section (lines 110-117) and acknowledged the potential for residual misclassification in the Limitations section (lines 343-344).

Comment 3: *"Given the modest AUCs, emphasize that these indices are not yet suitable for standalone clinical prediction."*

Response 3: We agree with the reviewer and thank them for this valuable suggestion. We have revised the Results (lines 243-246), Discussion (lines 258-262), and Conclusion (lines 359-361) sections to explicitly state that these indices are not suitable as standalone tools for infertility prediction in women with PCOS and should be interpreted as adjunctive metabolic markers rather than independent screening instruments.

Comment 4: *"Expand limitations: Address potential unmeasured confounders (e.g., diet, physical activity, detailed infertility workup)."*

Response 4: We have expanded the Strengths and Limitations section (lines 344-348) to explicitly acknowledge the potential influence of unmeasured confounders (as you asked for diet, physical activity, and detailed infertility workup) and their possible role in the residual confounding.

Comment 5: *"Consider adding a sensitivity analysis: Excluding women with very high TG-HDL ratios (>2.5) where the association became non-significant."*

Response 5: We thank the reviewer for this insightful suggestion. To address potential non-linearity and the influence of extreme values, we modeled the association between the TG–HDL ratio and infertility using restricted cubic spline analysis, which provides a flexible and statistically appropriate assessment without imposing arbitrary cut points. The spline analysis demonstrated a significant overall and non-linear association, with loss of statistical significance at higher TG–HDL values.

Importantly, as illustrated in the spline plot (barcode plot on the X axis of Fig. 1), the number of observations decreases substantially at TG–HDL ratios above approximately 2.5, which may partly explain the widening confidence intervals and loss of statistical significance in this range, rather than a true absence of association.

Given that non-linearity has already been formally evaluated and that post hoc exclusion based on a threshold may introduce selection bias, we chose not to perform an additional sensitivity analysis at this stage. However, if the reviewer or editor considers such an analysis essential, we would be pleased to perform it and report the results in a subsequent round of revisions or as supplementary material.

Comments on Figure 1:

Comment 1: Clarity of Red Dashed Line: The caption should explicitly state that the red dashed line represents OR = 1 (no association).

Response 1: We have revised the caption of Figure 1 accordingly (lines 230-233).

Comment 2: Threshold Annotation: Adding a vertical dotted line at TG-HDL ≈ 2.5 with a brief note (e.g., “threshold”) would help readers quickly interpret the inflection point.
Response 2: We have revised Figure 1 accordingly.

Comments on Figure 2:

Comment 1: “*The legend lists “TG_HDL” and “TyG_HDL.” However“TyG_HDL” is not described in the text*.”

Response 1: We have removed the TyG-HDL from Figure 2.

Comment 2: “*Some lines are very close together (AIP, TG-HDL). Consider using dashed/dotted styles or clearer color differentiation.*”
Response 2: We have made necessary edits and replaced Fig 2 with a new plot.

Comment 3: “*It would be helpful to add AUC values in the legend (e.g., “AIP (AUC = 0.625)”) for immediate comparison.*”
Response 3: We have added the AUC values in the new plot.

Comment 4: “*BMI” is included in the ROC but not discussed in the predictive performance results. Either explain its inclusion or remove it to avoid distraction.*”
Response 4: We have removed the BMI from the new plot.

Reviewer #2

Thank you for taking the time to review our work. We’re grateful for your guidance throughout the review process.

Comment 1: “*Authors failed to picture the rationality of this study? What is main novelity? Previous study answer this question how you fill the gap?*”

Response 1: The novelty of our study lies in the comprehensive, PCOS-specific evaluation of multiple metabolic indices, including the first assessment of AIP in relation to infertility, along with non-linear modeling and ROC analysis to assess clinical relevance. While we mentioned these in the last paragraph of our Introduction section, we have revised the text (lines 79-89) to more explicitly highlight these gaps and to clarify the novelty of our study.

Comment 2: "*How many women you excluded due to the exclusion criteria?*"

Response 4: We have clarified the number of participants excluded (n=335) based on the predefined exclusion criteria in the Study Design and Participants section (lines 160-161).

Comment 3: "*How were the techniues of assessments , kits, methodes…*"

Response 4: We have revised the Measurements and Tools section to provide additional details on the assessment techniques and methods (lines 103-109).

Comment 4: "*How was diagnostic criteria of PCOS?"*

Response 4: We have clarified the diagnostic criteria of PCOS in the Measurements and Tools section (lines 118-122).

Comment 5: "*What is definition of infertility?"*
Response 5: We have clarified the definition of infertility in the Measurements and Tools section (lines 110-117).

Comment 6: "*It was better to have a control group for comparison."*
Response 6: The primary objective of this study was to examine the association between metabolic indices and infertility within a population of women with PCOS, rather than to compare women with and without PCOS. Accordingly, we used an internal comparison between infertile (cases) and fertile (control) women with PCOS to minimize confounding related to the diagnosis of PCOS itself and to better characterize metabolic heterogeneity within this high-risk group.

While inclusion of a non-PCOS control group may be informative for other research questions, it was beyond the scope of the present study.

Comment 7: "*Please discuss about any pollsible bias of study."*
Response 7: We have expanded the Strengths and Limitations section to explicitly discuss potential sources of bias(including selection bias related to the tertiary-care setting, residual confounding from unmeasured lifestyle and reproductive factors, etc.) (lines 344-351).

Comment 8: "*What is clinical implication of study?"*
Response 8: We have added a *Clinical Implications* subsection to the Discussion to clarify the potential relevance of our findings (lines 318-327).

Comment 9: "*Editor advised to check the usig of AI.*"

Response 9: We confirm that no artificial intelligence tools were used for data collection, statistical analysis, result generation, or scientific interpretation in this study. Any language editing, where applicable, was limited to improving grammar and clarity, and all content was reviewed and approved by the authors.

Sincerely,

Dr. Amene Abiri
